# Overcoming Energy Storage‐Loss Trade‐Offs in Polymer Dielectrics Through the Synergistic Tuning of Electronic Effects in *π*‐Conjugated Polystyrenes

**DOI:** 10.1002/advs.202415738

**Published:** 2025-01-30

**Authors:** Yipin Cheng, Honghong Gong, Meirong Zhang, Qinglong Ji, Guanxiang Zhang, Xiao Zhang, Zhicheng Zhang

**Affiliations:** ^1^ Department of Applied Chemistry Xi'an Key Laboratory of Sustainable Energy Materials Chemistry National Innovation Platform (Center) for Industry‐Education Integration of Energy Storage Technology Engineering Research Center of Energy Storage Materials and Devices of Ministry of Education School of Chemistry Xi'an Jiaotong University Xi'an 710049 P. R. China; ^2^ National Key Laboratory of Science and Technology on Vessel Integrated Power System Naval University of Engineering Wuhan 430034 P. R. China

**Keywords:** electron cloud distortion, electron conduction, electron injection, electronic effects

## Abstract

Achieving high‐performance dielectric materials remains a significant challenge due to the inherent trade‐offs between high energy storage density and low energy loss. A central difficulty lies in identifying a suitable dipolar unit that can enhance the polarity and dielectric constant of the material while effectively suppressing the high energy losses associated with polarization relaxation, charge injection, and conduction. To address this, a novel strategy is proposed that introduces electron‐donating and electron‐withdrawing substituents on the benzene ring of polystyrene‐based polymers, creating bulky dipole groups that are resistant to reorientation under an electric field. This approach mitigates relaxation losses associated with dipole reorientation and manipulates the band structure via substituent modification to suppress conduction losses. Additionally, the deformation of the *π*‐electron cloud under an electric field enhances the dielectric constant and energy storage density. Ultimately, the optimized chlorostyrene‐methyl methacrylate (MMA) copolymer exhibits an 85% discharge efficiency and an energy storage density of 18.3 J cm^−^^3^, nearly three times that of styrene‐based copolymers under the same conditions. This study introduces a new approach for designing high‐energy density, low‐loss polymer dielectric materials by precisely controlling electron‐donating and electron‐withdrawing effects to modulate the distribution of *π*‐conjugated electron clouds.

## Graphical abstract

The bulky side groups confer the dielectric with exceptional dielectric stability and very low relaxation losses. The electron‐withdrawing effects enhance the response of the electron cloud to the electric field and suppress electron injection, while the electron‐donating effects increase the intrinsic polarity and inhibit electron conduction, thereby overcoming the trade‐off between high energy storage and low energy loss.

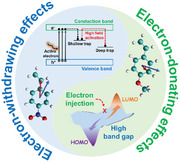

## 1 Introduction

Polymer dielectrics are a class of materials capable of isolating current under high electric fields while generating surface‐induced charges through polarization, thereby enabling energy storage and discharge.^[^ ^1^ ^]^ Due to their excellent film‐forming properties, low loss, and high breakdown strength, polymer dielectrics are widely employed in the manufacturing of thin‐film capacitors, serving as essential components in power systems, household appliances, electrical grids, and electric vehicles.^[^ ^2^ ^]^ However, with the increasing demand for high energy density capacitors in sectors such as electromagnetic energy devices, the most commercially used biaxially oriented polypropylene (BOPP) films face significant challenges. Their low dielectric constant (*ε*_r_ ≈2.2), stemming from low polarity, fails to meet the requirements of modern high‐energy density capacitors. Moreover, increasing the breakdown strength (*E*_b_) to enhance energy storage density significantly elevates the risk of capacitor breakdown, compromising long‐term safety and stability.^[^ ^3^ ^]^

To improve the energy storage density of polymer dielectrics, increasing the polarity of polymers to enhance their dielectric constant has become a widely adopted strategy. However, despite decades of research exploring various methods to increase polymer polarity, results have been limited. Typically, increasing the dielectric constant is accompanied by heightened energy loss, which not only hinders efficient energy discharge but also increases the risk of dielectric breakdown.^[^ ^4^ ^]^ One common approach involves introducing polar groups to enhance the dielectric constant through dipole reorientation under an external electric field.^[^ ^5^ ^]^ Unfortunately, the reorientation of polar groups often requires extended time, leading to significant polarization relaxation losses.^[^ ^6^ ^]^ Another method incorporates heterogeneous fillers to enhance interfacial polarization, but this approach frequently introduces interfacial losses and electric field distortions, making it difficult to balance high energy storage with low energy loss.^[^ ^7^ ^]^ Additionally, manipulating the polarization behavior of high‐polarity ferroelectric phases can yield high dielectric constants and energy densities, however, the longer relaxation times of large‐scale ferroelectric domains result in losses far greater than those of typical polar covalent bonds, exacerbating the problem of high energy loss.^[^ ^3^, ^6^, ^8^ ^]^ Recent strategies involving the introduction of small amounts of *π*‐conjugated units to construct charge‐trapping sites in polymers have shown promise in reducing conduction losses, but the absence of polar groups limits their contribution to energy storage density and fails to address the issue of relaxation losses.^[^ ^6^, ^9^ ^]^ Overall, existing polymer dielectric materials face a fundamental challenge in simultaneously achieving high energy storage density and low energy loss.^[^ ^10^ ^]^ The key issue lies in identifying a suitable dipolar unit that can enhance material polarity and dielectric constant while effectively suppressing energy losses caused by polarization relaxation, charge injection, and charge conduction.

To address this challenge, we propose a novel strategy of introducing electron‐donating and electron‐withdrawing substituents onto the benzene ring of polystyrene‐based polymers to construct bulky dipole groups that resist reorientation under an electric field. On one hand, this approach leverages the deformation of the *π*‐electron cloud of the benzene ring under the influence of an electric field to enhance the dielectric constant and energy storage density. On the other hand, it avoids the relaxation losses caused by dipole reorientation and modifies the band structure by substituents to suppress conduction losses. Ultimately, this strategy enables the simultaneous achievement of high energy storage density and low energy loss in polymeric dielectrics. We systematically calculated the dipole moments and electron localization functions (ELF) of styrene monomers with different substituents and selected three styrene monomers with nitro, chlorine, and methoxy substituents for polymerization.^[^ ^11^ ^]^ Through a fair comparison, we studied the effects of dipole moment magnitude and ELF bifurcation values on the dielectric and energy storage properties of the resulting polymers. The results demonstrated that the chlorinated styrene monomer exhibited a balanced dipole deformation capability and band structure, enabling both high energy storage density through increased polarity and effective suppression of relaxation and conduction losses. This superior performance was observed in both homopolymers and copolymers with methyl methacrylate (MMA), outperforming other monomers in terms of energy storage and loss characteristics. The optimized chlorostyrene copolymer exhibited a discharge efficiency of 85% and an energy storage density of 18.3 J cm^−^^3^, nearly three times that of polystyrene‐based copolymers under the same conditions. This study introduces a novel approach for designing high‐energy density, low‐loss polymer dielectric materials through precise control of electron‐donating and electron‐withdrawing effects to manipulate the distribution of *π*‐conjugated electron clouds.

## 2 Results and Discussion

### 2.1 Influence of Electronic Effects on the Microscopic Structure

The electronic effects of substituents significantly influence the electronic activity of the benzene ring, and alter the behaviors of conjugated electron clouds.^[^ ^9f^ ^]^ Clarifying the impact of these effects on the behavior of the electron cloud is essential for achieving high‐performance dielectrics. Employing density functional theory (DFT), the electron‐withdrawing and electron‐donating effects of substituents on styrene are assessed by analyzing the dipole moment (µ) and the isosurface of the electron localization function (ELF‐*π*).^[^ ^12^ ^]^ To facilitate synthesis and large‐scale preparation, 9 representative *p*‐substituted styrene‐bearing compounds are examined by calculating their dipole moments and ELF‐*π* bifurcation value between the substituents and the benzene ring as summarized in **Table** **1**. Additionally, by calculating the molecular volume, we accounted for spatial effects to ensure the accuracy of our experimental conclusions.

**Table 1:** Typical *p*‐substitution properties of styrene.

Entry	Dipole Moment^a)^ [Debye]	Isosurface Fracture Value^b)^	Molecular Volume^c)^ [cm^3^ mol^−1^]
St‐NO_2_	4.96	0.14	106.04
St‐CN	4.52	0.17	103.73
St‐COH	3.34	0.20	104.35
St‐CF_3_	3.02	< 0.10	107.26
St‐Cl	2.43	0.12	105.28
St‐NH_2_	2.98	0.39	99.02
St‐OCH_3_	1.87	0.30	107.05
St‐OH	1.64	0.30	95.46
St‐CH_3_	0.61	0.19	103.39

^a)^ The dipole moment of the monomer is calculated by DFT simulation;

^b)^ ELF‐*π* isosurface fracture value is calculated by DFT simulation and Multiwfn;

^c)^ The molecular volume of the monomer is calculated by Marching Tetrahedron.

**Figure 1:**
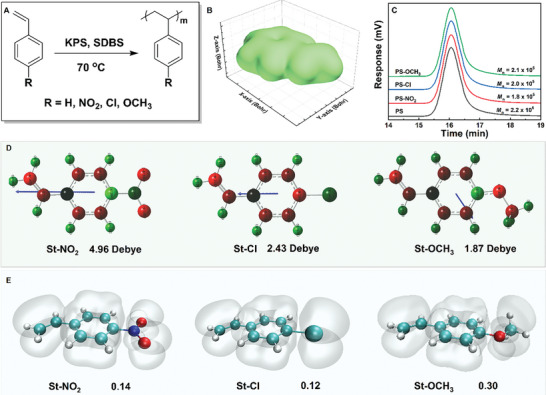
A) Schematic of polymer synthesis, B) molecular volume simulation diagram of PS, C) polymer molecular weight and distribution, D) molecular dipole moment, and E) ELF‐*π* isosurface.

Halogen‐substituted benzene, with balanced electron‐withdrawing and electron‐donating effects on benzene rings, is employed as a reference. As shown in **Figure** **1**A, among the 9 candidates, 4‐chlorostyrene (St‐Cl), 4‐nitrostyrene (St‐NO₂), and 4‐methoxystyrene (St‐OCH₃) are selected as target monomers to synthesize their corresponding polymers by free radical emulsion polymerization. The volumes of the substituted styrene molecules, approximately (106 ± 1) cm^3^ mol^−1^ (Table 1, Figure 1B), and the molecular weights of three sets of polymers, around (2.0 ± 0.2) × 10⁵ g mol^−1^ (Figure 1C; Table S1, Supporting Information) are consistently controlled. Hence, variations in polymer properties observed in subsequent electrical performance tests are anticipated to stem primarily from the electronic effects of the substituents.

Taking the chlorine atom as a reference, the distinct electronic effects of nitro and methoxy groups are explored as shown in Figure 1D,E. The ELF‐*π* bifurcation values of 0.14 for St‐NO_2_ and 0.12 for St–Cl (Figure 1D; Figure S1, Supporting Information) confirm their comparable electron‐donating abilities. However, St‐NO_2_’s dipole moment of 4.96 Debye is significantly higher than St‐Cl's 2.43 Debye, indicating a stronger electron‐withdrawing effect. Conversely, St‐OCH₃ exhibits a lower dipole moment of 1.87 Debye compared to St–Cl and a higher ELF‐*π* bifurcation point of 0.30, suggesting a significantly stronger electron‐donating effect. Therefore, the performance variations in PS‐NO_2_ should be predominantly influenced by its electron‐withdrawing effect, whereas those in PS‐OCH₃ should be primarily affected by its electron‐donating effect. These distinctions in polymer performance will offer valuable insights into the influence of electronic effects on the dielectric and insulating properties of polymers.

**Figure 2:**
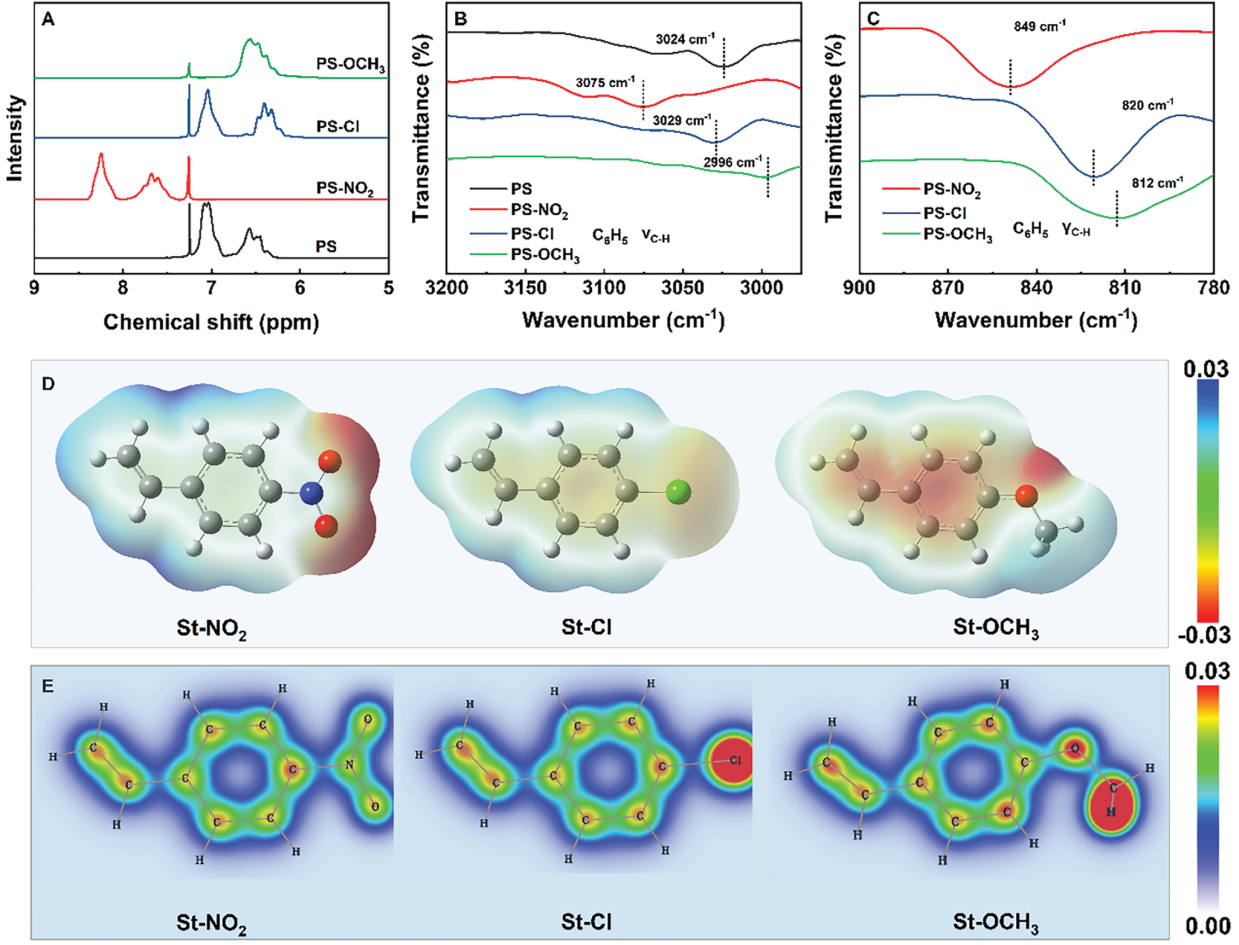
A) ^1^H NMR characterization of proton hydrogen, C–H B) stretching vibration peak, C) in‐plane bending vibration peak in the polymer benzene ring, and D) surface electrostatic potential distribution, E) *π*‐electron density distribution of the substituted monomer.

The chemical structures of the polymers are characterized using ^1^H NMR and FT‐IR, which confirm the influence of various electronic effects on the electron cloud of benzene ring. **Figures** **2**A and S2 (Supporting Information) display the proton signals at the *ortho*, *meta*, and *para* positions of the benzene ring, ranging from 6.0 to 8.5 ppm. The chemical environment of the benzene protons in PS‐Cl closely resembles that in PS, showing similar chemical shifts. The strong electron‐withdrawing effect of the nitro group causes a downfield shift in the benzene proton chemical shifts by altering the electron distribution.^[^ ^9f^ ^]^ Conversely, the strong electron‐donating effect of the methoxy group increases electron delocalization, leading to an upfield shift that merges the benzene proton signals into a single chemical shift.^[^ ^13^ ^]^ FT‐IR analysis corroborates these findings. Figures 2B,C and S3 (Supporting Information) illustrate the stretching and in‐plane bending vibration peaks of the benzene C─H bonds at 3029 and 820 cm^−1^ in PS‐Cl. In PS‐NO_2_, the strong electron‐withdrawing effect of nitro group causes a blue shift of the C─H bond vibration peaks to 3075 and 849 cm^−1^. In PS‐OCH_3_, the electron‐donating effect of methoxy group leads to *π*‐electron delocalization, resulting in a red shift of the C─H bond vibration peaks to 2996 and 812 cm^−1^. These modifications in the polymer chemical structures confirm that different electronic effects of substituents may dramatically alter the electron cloud distribution on the benzene ring.

The significant difference in the surface electrostatic potential of various substituted styrene vividly demonstrates the impact of electronic effects on the electron cloud distribution of benzene rings. As shown in Figure 2D, the electron cloud in St‐NO_2_ shifts noticeably towards the nitro group, significantly reducing the electron cloud density on the benzene ring. In contrast, in St‐OCH_3_, the electron‐donating effect enhances the electron cloud activity, concentrating it on the benzene ring. The *π*‐electron density distribution further illustrates these differences. As shown in Figure 2E, compared to the balanced electronic effects in St–Cl, St‐NO_2_ exhibits higher electron density at the *ortho* position, while St‐OCH_3_ shows higher electron density at the *meta* position. In summary, electron‐withdrawing effects cause notable deformation in the benzene ring electron cloud, while electron‐donating effects enhance its activity. This regulation of electron cloud morphology and activity is crucial for designing polymer materials with specific dielectric properties.

### 2.2 Influence of Electronic Effects on the Dielectric Properties

**Figure 3:**
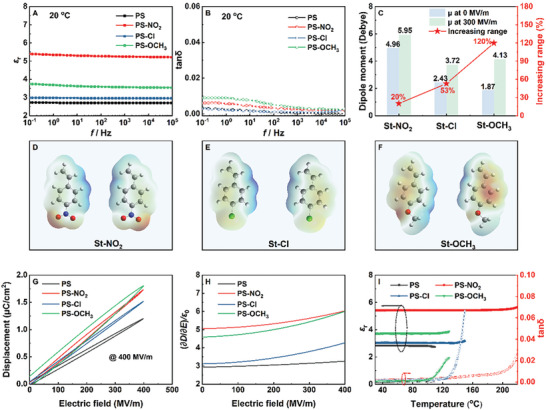
A) Dielectric constant, B) loss factor of the polymer as a function of frequency, C) average dipole moment of the molecule under different electric fields, D–F) surface electrostatic potential under opposing electric fields (the front and back are the same), G) *D‐E* loop of the polymer at 400 MV m^−1^, H) polarization strength under different electric fields, and I) variation of dielectric constant and loss factor as a function of temperature.

Unlike traditional polymers that achieve high polarization strength through orientation polarization accompanied by high relaxation loss, the bulky benzene rings in styrene‐based polymers resist flipping under an external electric field. Their polarization contribution mainly arises from electronic and ionic polarization. To explore the impact of substituent electronic effects on the dielectric properties of styrene‐based polymers, the dielectric constant and loss factor (tan δ) of the polymers are measured at different temperatures and frequencies. As shown in **Figure** **3**A,B, the polymers exhibit negligible changes in dielectric constant across the entire frequency range and maintain very low dielectric loss, demonstrating excellent dielectric stability. This indicates that the enhancement of the dielectric constant in styrene‐based polymers does not rely on traditional orientation polarization mechanisms, thereby avoiding the high relaxation loss typically associated with orientation polarization. This indicates the electronic effects and the polarization behavior of the benzene ring electron cloud are responsible for the different dielectric responses from the conventional glassy polymer dielectrics.

Although the introduction of polar groups increases the dielectric constant of the polymer, the change in the dielectric constant does not fully correspond to the change in the dipole moment. For example, compared to the dielectric constant of 2.70 for PS at 1 kHz, PS‐NO_2_ with the highest monomer dipole moment (4.96 Debye), has a dielectric constant of 5.26. Conversely, PS‐OCH_3_, despite having a higher dielectric constant (3.57) than PS‐Cl (2.96), has a dipole moment (1.87 Debye) lower than 2.43 Debye of St–Cl. Considering that electronic effects can alter the behavior of the benzene ring electron cloud, the average dipole moment of substituted styrene is further analyzed under an electric field (FiguresS4–S6 and Table S2, Supporting Information). As shown in Figure 3C, under a 300 MV m^−1^ electric field, the dipole moment of St‐OCH_3_ significantly increases, with an average dipole moment reaching 4.13 Debye, higher than 3.72 Debye of St–Cl. At this point, the change in dipole moment agrees perfectly with the change in dielectric constant. Notably, compared to the dipole moment without an electric field, the dipole moment of St‐OCH_3_ is increased by 120%, far exceeding the increases of 53% for St–Cl and 20% for St‐NO_2_. Simulations of the molecular surface electrostatic potential under an opposing electric field (Figure 3D–F) further confirm the differences in electron cloud changes. Combining the previous discussion, it can be inferred that although electron‐withdrawing effects can alter the electron cloud morphology and increase the inherent dipole moment, they also limit the response of the electron cloud against the electric field. In contrast, electron‐donating effects can activate the benzene ring electron cloud, enhance its response to the electric field thus achieve higher polarization strength.

To validate this hypothesis, the electirc displacment‐electric field (*D*‐*E*) loops of the polymers are measured under a 400 MV m^−1^ electric field (Figure 3G). The results indicate that the electric displacement of PS‐OCH_3_ exceeds that of PS‐NO_2_ and is significantly higher than that of PS‐Cl. Using the equation,

(1) $$D=\varepsilon_{0}\;\varepsilon_{r}E$$

the polarization strength is calculated under different electric fields. As shown in Figure 3H, at low electric fields, the variation in polarization strength aligns with the trends observed in the dielectric constant and dipole moment. With increasing electric field, the response of PS‐OCH_3_ surpasses PS‐Cl and is notably higher than PS‐NO_2_. Ultimately, under electric fields exceeding 400 MV m^−1^, the polarization strength of PS‐OCH_3_ even surpasses that of PS‐NO_2_. Additionally, the temperature dependence of the dielectric properties at 10^3^ Hz (Figure 3I) further verifies that the enhanced polarization strength of styrene‐based polymers relies on electron cloud deformation rather than orientation polarization. This study reveals, for the first time, the differential impact of various electronic effects on the benzene ring electron cloud and confirms the potential of styrene‐based polymers as dielectric materials.

### 2.3 Influence of Electronic Effects on the Insulation Properties

**Figure 4:**
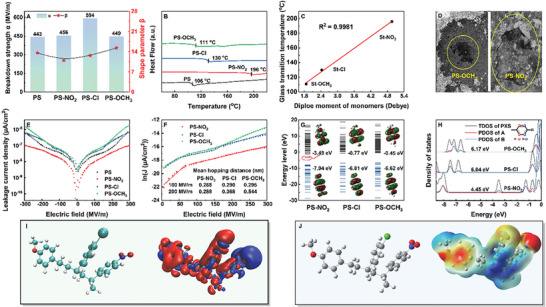
A) Weibull distribution statistics of breakdown strength, B) DSC characterization results, C) relationship between 𝑇_g_ and molecular dipole moment, D) crack image after breakdown, E) leakage current, F) fitted plot of hopping conductivity of polymers, and G) band structure of the structural unit, H) density of electronic states, I) electronic density difference, and J) surface electrostatic potential of the polymer.

Due to the conjugated benzene rings in styrene‐based polymers facilitating electron conduction under an electric field, their *E*_b_ is significantly lower than that of traditional polymers with low polarity. By applying a two‐parameter Weibull statistical distribution for precise analysis of the 𝐸_b_ of the polymers, the breakdown mechanisms of styrene‐based polymers are examined from multiple perspectives and the relationship between electronic effects and benzene ring electron conduction and injection is established. As shown in **Figures** **4**A and S7 (Supporting Information), the 𝐸_b_ of PS‐Cl reached 594 MV m^−1^, significantly higher than 456 MV m^−1^ for PS‐NO_2_, 449 MV m^−1^ for PS‐OCH_3_, and 443 MV m^−1^ for PS. In general, the change in *E*_b_ tends to be positively correlated with the glass transition temperature (*T*_g_) of the polymers.^[^ ^7d^ ^]^ The *T*_g_ increases progressively from PS‐OCH_3_ to PS‐Cl to PS‐NO_2_, corresponding to changes in monomer dipole moments (Figure 4B,C). The electron‐withdrawing effect enhances intermolecular interactions, thus ultimately increasing the *T*_g_ of the polymers. The reduced density gradient function (RDG) simulation results (Figure S8, Supporting Information) further verify the enhancement of weak interactions within the polymers, with substituted polymers showing a peak near zero on the X‐axis compared to PS, indicating the increased weak interactions and cohesive energy. Therefore, from the mechanical breakdown perspective, PS‐NO_2_ and PS‐OCH_3_ in substituted styrene‐based polymers should exhibit the highest and lowest *E*_b_, respectively. However, the testing results are rather different from that, indicating that the breakdown strength is influenced by multiple factors more than mechanical breakdown mode. Additionally, PS‐NO_2_ shows significant mechanical damage in defect areas, which may lead to increased capacity loss with each breakdown. This compromises the self‐healing ability of the insulating performance in metalized film capacitors, which relies on forming an insulating zone around the breakdown site through the evaporation of the conductive layer, thereby also reducing the material's *E*_b_.^[^ ^9g^ ^]^

Leakage current density is a crucial parameter for analyzing the 𝐸_b_ of polymers from both electrical and thermal breakdown perspectives, providing essential information on the conductive behavior of materials under different electric fields. As illustrated in Figure 4E, the leakage current density curves of the polymers exhibit a nonlinear three‐segment distribution under an increased electric field. In the range of 100–200 MV m^−1^, the current density of PS increases at a lower speed with the electric field, demonstrating good insulating performance. However, when the electric field exceeds 200 MV m^−1^, the current density sharply increases due to the conjugated structure of the benzene ring, which provides a conductive path for electron conduction at high fields, leading to a rapid increase in electron mobility. PS‐OCH_3_ exhibits a similar pattern, but the electron‐donating effect leads to the earlier end of the second segment change and increases the overall leakage current density significantly, indicating that the electron‐donating effect activates the electron cloud and increases leakage current. Conversely, PS‐NO_2_ with the electron‐withdrawing effect could reduce the leakage current by an order of magnitude and effectively suppress the third segment current change. Under an electric field exceeding 100 MV m^−1^, the increasing speed of current density is significantly reduced, indicating that the electron‐withdrawing effect effectively inhibits the increase of leakage current. By fitting experimental data using the hopping conduction mechanism equation,^[^ ^14^ ^]^

(2) $$\ln\left(J\right)=\frac{qa}{kT}\;L+\ln\left(qanv\right)-\frac{E_{a}}{kT}$$

the average hopping distance (*a*) under an external electric field can be derived. An increase in average hopping distance implies fewer deep traps. As shown in Figure 4F, PS‐NO_2_ has the shortest average hopping distance, indicating that the electron‐withdrawing effect favors the formation of charge traps. In contrast, the average hopping distance of PS‐OCH_3_ is higher than that of PS‐Cl and increases more significantly against the electric field, which can be attributed to the electron‐donating effect activating the electron cloud and hindering trap formation. Therefore, although the electron‐donating effect in PS‐Cl causes a higher leakage current, the electron‐withdrawing effect helps create deep traps and suppress the third segment current change, which successfully inhibits the rapid increase in leakage current under high fields.

Studying changes in polymer band structure and electron affinity elucidates the source of leakage current and validates related hypotheses. Figure 4G illustrates the energy level distribution of the benzene ring units of the polymers at 300 MV m^−1^, where the electron‐withdrawing effect lowers the lowest unoccupied molecular orbital (LUMO) energy level of the benzene ring units in PS‐NO_2_ from −0.77 eV in PS‐Cl to −3.49 eV. Density of states (DOS) analysis (Figure 4H) confirms that the reduction in the LUMO energy level in PS‐NO_2_ is primarily due to atoms near the nitro group, indicating that the electron‐withdrawing effect enhances electron affinity and electron trapping ability, effectively inhibiting electron conduction. To gain a better understanding of the impact of the electron‐rich benzene ring structure on electron transport behavior, we have supplemented the energy level structures of three repeating units, as shown in Figure S9 (Supporting Information). The LUMO energy level of PS‐OCH_3_ is 0.03 eV, which is significantly higher compared to PS‐Cl at −0.58 eV and PS‐NO_2_ at −2.77 eV, consistent with the results obtained for individual structures. The electron‐withdrawing effect contributes to the suppression of electron conduction, while the electron‐donating effect mitigates this suppression. Electron density difference analysis (Figure 4I) further corroborates this, with red areas indicating electron depletion and blue areas indicating electron accumulation under an external electric field. The results show that PS‐NO_2_ with higher electrophilicity than PS‐Cl, can attract a large number of electrons and act as a charge trap under an electric field, inhibiting electron conduction. In contrast, the LUMO energy level of benzene ring units in PS‐OCH_3_ increases to −0.45 eV, suggesting that the electron‐donating effect leads to an electron‐rich benzene ring structure, facilitating the formation of conduction paths and promoting electron conduction. The electron conduction of styrene‐based polymers resembles that of semiconductor polymers, where a lower band gap leads to higher injected electron concentrations. Despite the slightly larger unit volume of PS‐OCH_3_ compared to PS‐Cl, it exhibits the highest band gap (6.17 eV), significantly higher than that of PS‐NO_2_ (4.45 eV). This indicates that the electron‐donating effect, by activating the electron cloud, exhibits strong electron‐rich characteristics capable of inhibiting electron injection, whereas the electron‐withdrawing effect enhances the electron affinity of the unit, aiding charge injection. Surface electrostatic potential maps (Figure 4J) further show that St‐OCH_3_ has more active electrons than St–Cl and St‐NO_2_, serving as primary evidence for increasing the band gap to inhibit electron injection. These findings provide new insights into the conduction mechanism of polymers and aid in designing dielectric materials with superior insulating performance and breakdown strength.

### 2.4 Influence of Electronic Effects on the Energy Storage Performance

**Figure 5:**
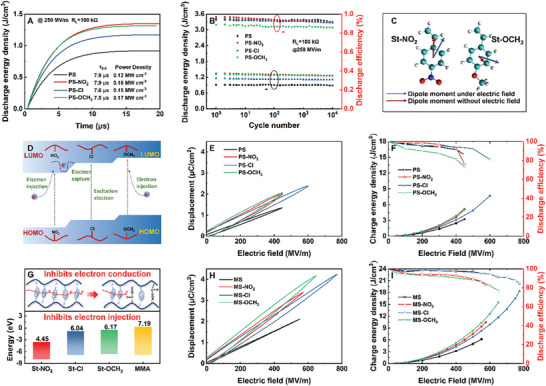
A) Charge–discharge performance, B) cyclic stability of polymer at 250 MV m^−1^, and C) schematic of molecular polarity changes under an electric field, D) schematic of molecular electron injection and conduction, E) *D‐E* loop, F) charge energy density and discharge efficiency of the styrene‐based polymer at maximum electric, G) electron injection and conduction, and band gap schematic of copolymer with methyl methacrylate, H) *D‐E* loop field, and I) charge energy density and discharge efficiency of the copolymer with methyl methacrylate at maximum electric.

Charge–discharge performance is a crucial indicator for evaluating the practical application of dielectric materials. **Figure** **5**A shows the test results of a 15 µm polystyrene‐based dielectric film under a 250 MV m^−1^ electric field. The electron‐donating effect activates the electron cloud, allowing for a rapid response to external electric fields, resulting in higher discharge density and the fastest discharge rate. In contrast, the electron‐withdrawing effect achieves the highest intrinsic dipole moment while suppressing the electron cloud response, leading to a slower discharge rate. Consequently, compared to PS, PS‐Cl utilizes both electron‐donating and electron‐withdrawing effects, increasing its power density by 25% over PS, reaching 0.15 MW cm^−^^3^. To verify the impact of electronic effects on the long‐term reliability of dielectric films, a rapid discharge cycling experiment was conducted under a 250 MV m^−1^ electric field (Figure 5B). PS‐NO_2_ exhibits poor cycling stability due to strong intermolecular forces caused by the electron‐withdrawing effect, increased modulus, and poor self‐healing, which are unfavorable for cyclic use. Conversely, the electron‐donating effect improves electric field response capability, reduces polymer modulus, and enhances self‐healing. PS‐OCH_3_ and PS‐Cl films demonstrated the best stability during 10⁴ charge–discharge cycles, making them particularly suitable for rapid charge–discharge needs under AC fields.

In summary, styrene‐based polymers exhibit excellent dielectric stability and extremely low relaxation loss due to the non‐flipping characteristics of the benzene ring structure under external electric fields, establishing a solid foundation for their application in dielectric materials. As shown in Figure 5C,D, introducing substituent groups with different electronic effects can effectively improve the dielectric and insulation properties of polystyrene‐based dielectrics. The electron‐withdrawing effect significantly enhances the polarization strength of the polymer by increasing the intrinsic dipole moment of styrene, improving the modulus, and inhibiting electron conduction, thus increasing its breakdown strength. However, high electron affinity may lead to increased electron injection, and strong interactions may cause mechanical damage, which are detrimental to improving the *E*_b_ and discharge efficiency of the polymer. The electron‐donating effect activates the electron cloud of the benzene ring, enhancing its response capability under an electric field, resulting in higher polarization strength at high fields. Moreover, the electron‐rich structure inhibits electron injection, further enhancing the *E*_b_. Nevertheless, the activated electron cloud induced by the electron‐donating effect may also promote electron conduction, increasing the risk of thermal and electrical breakdown, and increased energy loss at high fields. Therefore, in designing and optimizing polystyrene‐based dielectric materials, it is necessary to balance the electron‐withdrawing and electron‐donating effects, regulate the morphology and response capability of the styrene electron cloud, and control electron conduction and injection to achieve optimal dielectric and insulation properties.

By precisely controlling the type and content of substituent groups in styrene and adjusting the morphology of electron cloud and conduction characteristics, dielectric materials with high energy storage density and low loss can be achieved. As shown in Figure 5E and Figures S10–S13 (Supporting Information), the polarization strength of the polymer significantly increases, benefiting from both electron‐withdrawing and electron‐donating effects. However, the electron‐withdrawing effect promotes electron injection, while the electron‐donating effect facilitates electron conduction, both of which reduce *E*_b_ and discharge efficiency, and limit the increase in energy storage density. In contrast, PS‐Cl, with balanced electron‐withdrawing and electron‐donating effects, successfully suppresses electron injection and conduction, significantly enhancing the *E*_b_ of the polymer. Utilizing its high intrinsic polarity and electronic response capability, the energy storage performance is substantially improved. (Figure 5F). Additionally, the unique response mechanism and electron conduction injection capability of styrene‐based polymers indicate promising prospects for further improvement. For instance, the issue of electron conduction and injection can be further addressed by copolymerizing high band‐gap units. Poly(styrene‐methyl methacrylate) (MS), with a styrene monomer content of 53 mol%, was prepared by emulsion polymerization (Figures S14 and S15, Supporting Information). The high band gap and high polarity MMA units isolated the benzene ring structure and suppressed electron conduction. As shown in Figure 5G,H, the introduction of MMA units helps suppress electron injection, achieving synergistic improvement in breakdown strength, polarization strength, and energy loss. As illustrated in Figures 5I and S16 (Supporting Information), MS‐Cl exhibits a more than 103% increase in polarization strength, a 40% increase in breakdown strength (up by 225 MV m^−1^), and an 85% discharge efficiency, ultimately achieving an energy storage density of 18.3 J cm^−^^3^, a 197% improvement over MS.

## 3 Conclusion

This study introduces a novel strategy to reconcile the trade‐off between high energy storage density and low energy loss under strong electric fields in polymer dielectrics by incorporating electron‐withdrawing substituents to induce the formation of bulky dipoles in benzene rings. The bulky dipoles mitigate relaxation losses typically associated with traditional dipole reorientation, and the effect of the substituent modulates the electric field response of the *π*‐conjugated electron cloud and regulates charge conduction and injection behaviors. Chlorinated styrene monomers exhibit a well‐balanced dipole deformation capability and band structure, facilitating high energy storage density through enhanced polarity while effectively suppressing relaxation and conduction losses. This superior performance was observed both in homopolymers and in copolymers with MMA, while the optimized copolymer demonstrated a discharge efficiency of 85% and an energy storage density of 18.3 J cm^−^^3^, nearly three times that of styrene‐based copolymers under the same conditions. This study introduces a precise control strategy leveraging electron‐donating and electron‐withdrawing effects to manipulate the distribution of conjugated electron clouds, offering an innovative design approach for the development of polymer dielectric films with high energy storage and low energy loss.

## Conflict of Interest

The authors declare no conflict of interest.

## Author Contributions

The manuscript was written through the contributions of all authors. All authors have approved the final version of the manuscript. Yipin Cheng and Honghong Gong are co‐first authors.

## Supporting information



Supporting Information

## Data Availability

The data that support the findings of this study are available from the corresponding author upon reasonable request.
