# Supplementary material for: Overcoming Energy Storage‐Loss Trade‐Offs in Polymer Dielectrics Through the Synergistic Tuning of Electronic Effects in π‐Conjugated Polystyrenes
Source: Adv Sci (Weinh). 2025 Jan 30;12(11):2415738. doi: 10.1002/advs.202415738 (PMC11923881; doi:10.1002/advs.202415738)
Supplement: Supplementary file 1 — Supporting Information [file ADVS-12-2415738-s001.docx]

Supporting Information
©Wiley-VCH 2021
69451 Weinheim, Germany

Overcoming Energy Storage-Loss Trade-offs in Polymer Dielectrics through the Synergistic Tuning of Electronic Effects in π-Conjugated Polystyrenes

Yipin Cheng^[a]^, Honghong Gong*^[a]^, Meirong Zhang^[a]^, Qinglong Ji^[a]^, Guanxiang Zhang^[b]^, Xiao Zhang ^[b]^ and Zhicheng Zhang*^[a]^

**Abstract:** Achieving high-performance dielectric materials remains a significant challenge due to the inherent trade-offs between high energy storage density and low energy loss. A central difficulty lies in identifying a suitable dipolar unit that can enhance the polarity and dielectric constant of the material while effectively suppressing the high energy losses associated with polarization relaxation, charge injection, and conduction. To address this, we propose a novel strategy that introduces electron-donating and electron-withdrawing substituents on the benzene ring of polystyrene-based polymers, creating bulky dipole groups that are resistant to reorientation under an electric field. This approach mitigates relaxation losses associated with dipole reorientation and manipulates the band structure via substituent modification to suppress conduction losses. Additionally, the deformation of the π-electron cloud under an electric field enhances the dielectric constant and energy storage density. Ultimately, the optimized chlorostyrene-methyl methacrylate (MMA) copolymer exhibits an 85% discharge efficiency and an energy storage density of 18.3 J cm^-^³, nearly three times that of styrene-based copolymers under the same conditions. This study introduces a new approach for designing high energy density, low loss polymer dielectric materials by precisely controlling electron-donating and electron-withdrawing effects to modulate the distribution of π-conjugated electron clouds.

DOI: 10.1002/anie.2021XXXXX

Table of Contents

[**Experimental Procedures 2**](#_Toc139641559)

[**Chemicals and Materials 2**](#_Toc139641560)

[**Synthesis of polymer 2**](#_Toc139641561)

[**Fabrication of polymer films 2**](#_Toc139641562)

[**Instruments and characterization 2**](#_Toc139641563)

[**Results and Discussion 3**](#_Toc139641564)

[**Author Contributions 8**](#_Toc139641565)

Experimental Procedures

**Chemicals and Materials**

4-chlorostyrene (St-Cl, Shanghai Kaisai Chemical Co. Ltd, AR grade), 4-nitrostyrene (St-NO₂, Shanghai Macleane Biochemical Technology Co. Ltd, AR grade), and 4-methoxystyrene (St-OCH₃, Shanghai Macleane Biochemical Technology Co. Ltd, AR grade), methyl methacrylate (MMA, Shanghai Macleane Biochemical Technology Co. Ltd, AR grade) were washed twice with 5% NaOH aqueous solution and deionized water, respectively. The obtained monomers were subsequently dried overnight with MgSO_4_, distilled under reduced pressure, and stored under N_2_ at a temperature of 10 ^o^C. Potassium persulfate (KPS, Tianli Reagents Co. Ltd, AR grade), sodium dodecyl benzene sulfonate (SDBS, Meryer (Shanghai) Chemical Technology Co. Ltd, AR grade), *N*, *N*-dimethylformamide (DMF) and methanol (MeOH) were purchased from Tianjin Reagents Co. Ltd and used as received.

**Synthesis of polymer**

The standard protocol for the production of polystyrene (PS) is illustrated in **Figure 1A**. Wherein, 5 wt% of SDBS was introduced into a 100 mL Schlenk bottle followed by three cycles of vacuum-nitrogen filling in order to eliminate any oxygen and moisture. St of 5 mL was dispersed in water before being transferred to the Schlenk bottle and placed in an oil bath. The polymerization was conducted at 70 ^o^C for 24 h once the pre-emulsification was completed through vigorous stirring and 5 mL of water containing 1 wt% KPS was added to the reaction mixture in 30 minutes. 15 mL of saturated saline was added dropwise to terminate the polymerization and break the emulsion. The precipitate was collected by filtration and purified by repeatedly dissolving in DMF followed by precipitating in water and methanol three times. Finally, the product was dried under reduced pressure at 40 ^o^C for 12 h before characterization. All polymers were synthesized following the same method for comparison purposes.

**Fabrication of polymer films**

Polymer films were fabricated following a solution-casting process. The polymers were dissolved in DMF with a concentration of 2 wt%, and the solutions were deposited onto glass slides followed by subjected to a temperature of 65 ^o^C. After the solvent was completely evaporated, the films along with the slides were annealed at 170 ^o^C for 4 h prior to analysis. The thickness of all films is about 11-13 μm.

**Instruments and characterization**

^1^H NMR spectra were recorded on a Bruker spectrometer (400 MHz, Advance III) in CDCl_3_ with tetramethylsilane (TMS) as an internal standard. FT-IR spectroscopy was performed on a Tensor27 (Bruker, Germany) in transmittance mode with a resolution of 1 ~ 0.4 cm^-1^. Molecular weights and polydispersity index (PDI) were determined using a Viscotek GPCmax autosampler system consisting of a pump, ViscoGEL GPC columns (G4000H HR), and a Waters 2414 differential refractive index (RI) detector with a DMF flow rate of 1.0 mL min^-1^ at 30 ^o^C. DSC thermograms were obtained on a Netzsch DSC 200 PC (Netzsch, Germany) under a nitrogen atmosphere at a scanning rate of 10 ^o^C min^-1^ after a cycle of heating (10 ^o^C min^-1^) and cooling (10 ^o^C min^-1^) to remove the thermal history. The variation of the modulus of the polymer with temperature was characterized using Netzsch DMA242E. The surface morphology of polymers was detected with a tungsten hairpin filament scanning electron microscope (ZEISS EVO 10), where the films were coated with a thin layer of gold before observation. Gold electrodes were sputtered on both surfaces of the polymer films on a JEOL JFC1600 autofine coater (Japan). Broadband dielectric spectroscopy (BDS) measurements were performed on a Novocontrol Concept 80 broadband dielectric spectrometer with temperature control. The applied voltage was 1 V with the frequency changing from 0.1 Hz to 1 MHz at different temperatures. The electric displacement-electric field (*D*-*E*) loops at room temperature were obtained on a Premiere II ferroelectric tester from Radiant Technologies, Inc., where AC electric fields ranging from 50 ~ 850 MV m^-1^ were applied across polymer films with a triangular waveform at a frequency of 10 Hz. The *E*_b_ for all the sample films was determined using a breakdown voltage tester (Beijing Beiguangjingyi Instrument Equipment Co., Ltd), and the electric field was applied at a rate of 1 kV min^-1^. The diameter of the cylindrical electrode was 10 mm. A high-voltage DC power supply (RK2674B) was used to evaluate *E*_b_ of the films. In fast discharge experiments, films were charged to a given voltage using a high voltage MOSFET switch (610D-K-CE-IX); the inner resistance (RL) was 100 kΩ, and the discharge curve was recorded under varied electric fields. The leakage current was obtained as less than 10 mA until the film was electrically broken down. Density functional theory (DFT) calculations were performed with the Gaussian 09 with B3LYP hybrid function and the 3-21 G basis function. The analysis of reduced density gradient (RDG) was performed within the Multiwfn program

Results and Discussion

**Table S1.** PFSMs and PSM with varied compositions are synthesized.

| Entry^[a]^ | Mn^[c]^ (× 10^5^ g mol^-1^) | PDI |
| --- | --- | --- |
| PS | 2.2 | 1.14 |
| PS-NO_2_ | 1.8 | 1.21 |
| PS-Cl | 2.0 | 1.18 |
| PS-OCH_3_ | 2.1 | 1.17 |

[a] 5 mL water, 0.05 g SDBS, and 0.01 g K_2_S_2_O_8_ are utilized and the reactions are conducted at 70 ^o^C. [b] The molar ratio is determined by ^1^H NMR. [c] Molecular weight and PDI is determined by GPC.


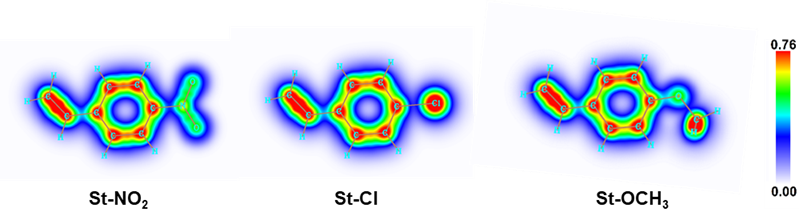


**Figure S1.** The LOL-π planar graph of molecule.


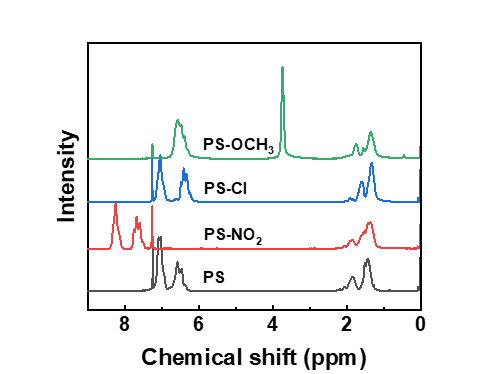


**Figure S2.** The ^1^H NMR characterization of polymers.


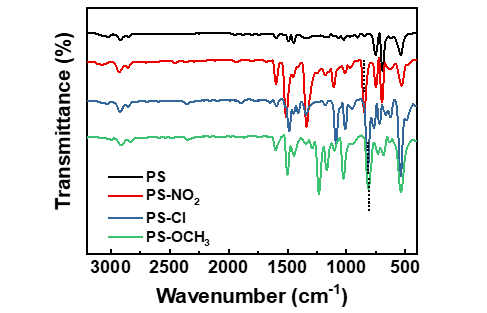


**Figure S3.** The FT-IR spectra of polymers.


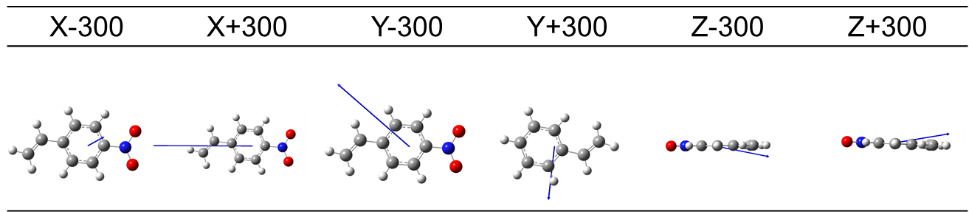


**Figure S4.** The average dipole moment of PS-NO_2_ under a 300 MV/m electric field.


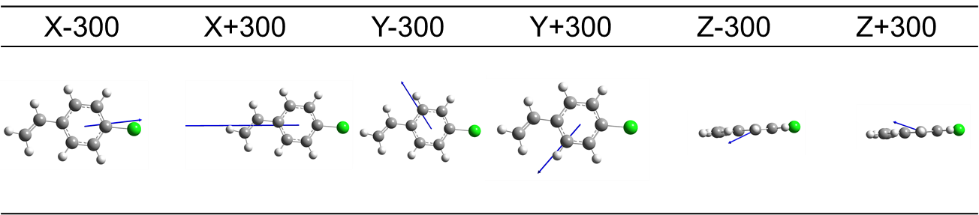


**Figure S5.** The average dipole moment of PS-Cl under a 300 MV/m electric field.


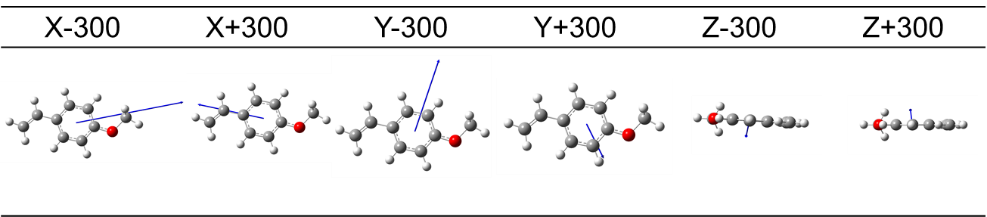


**Figure S6.** The average dipole moment of PS-OCH_3_ under a 300 MV/m electric field.

**Table S2.** Dipole moment of XSt under electric field.

| **Entry** | **X-300** | **X+300** | **Y-300** | **Y+300** | **Z-300** | **Z+300** | **Aver.** |
| --- | --- | --- | --- | --- | --- | --- | --- |
| St-NO_2_ | 1.2560 | 11.9725 | 6.1555 | 6.2446 | 5.0430 | 5.0430 | 5.9524 |
| St-Cl | 4.2061 | 4.2319 | 4.2319 | 4.2061 | 2.7342 | 2.7342 | 3.7241 |
| St-OCH_3_ | 8.0529 | 4.9122 | 5.0135 | 2.4036 | 2.1998 | 2.1998 | 4.1303 |

[a] The dipole moment is obtained by DFT calculation.


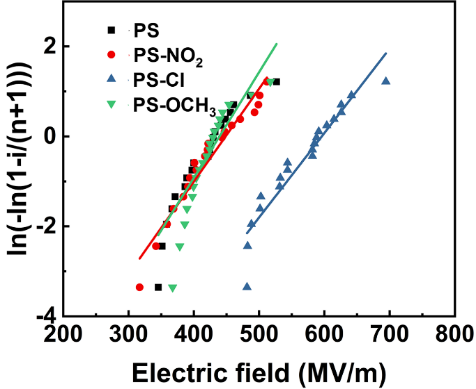


**Figure S7.** The breakdown strength of polymers at room temperature.


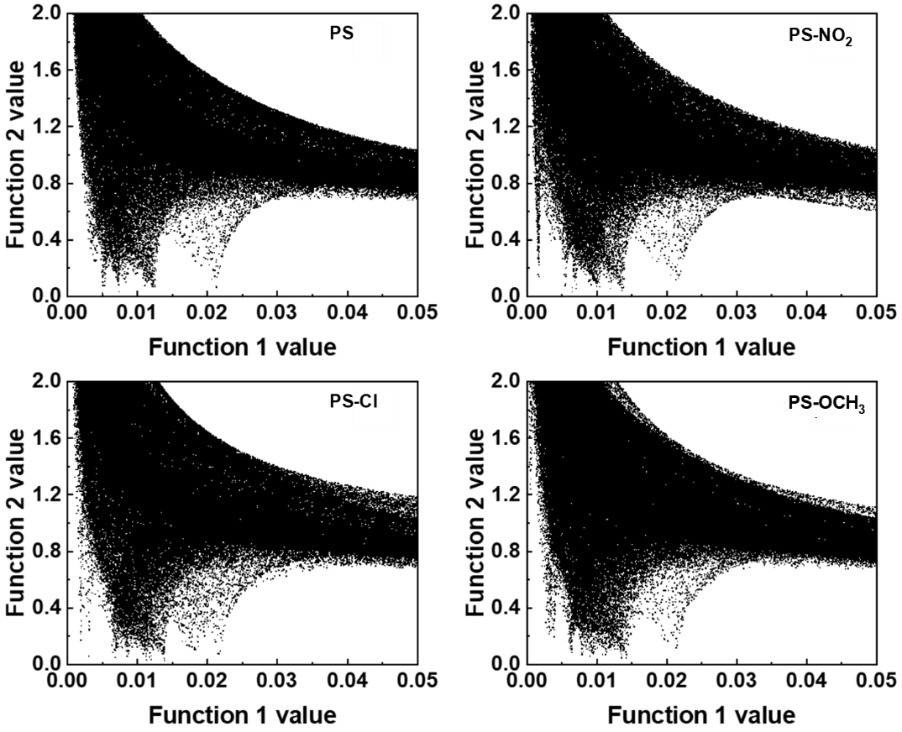


**Figure S8.** The RDG simulation of polymers.


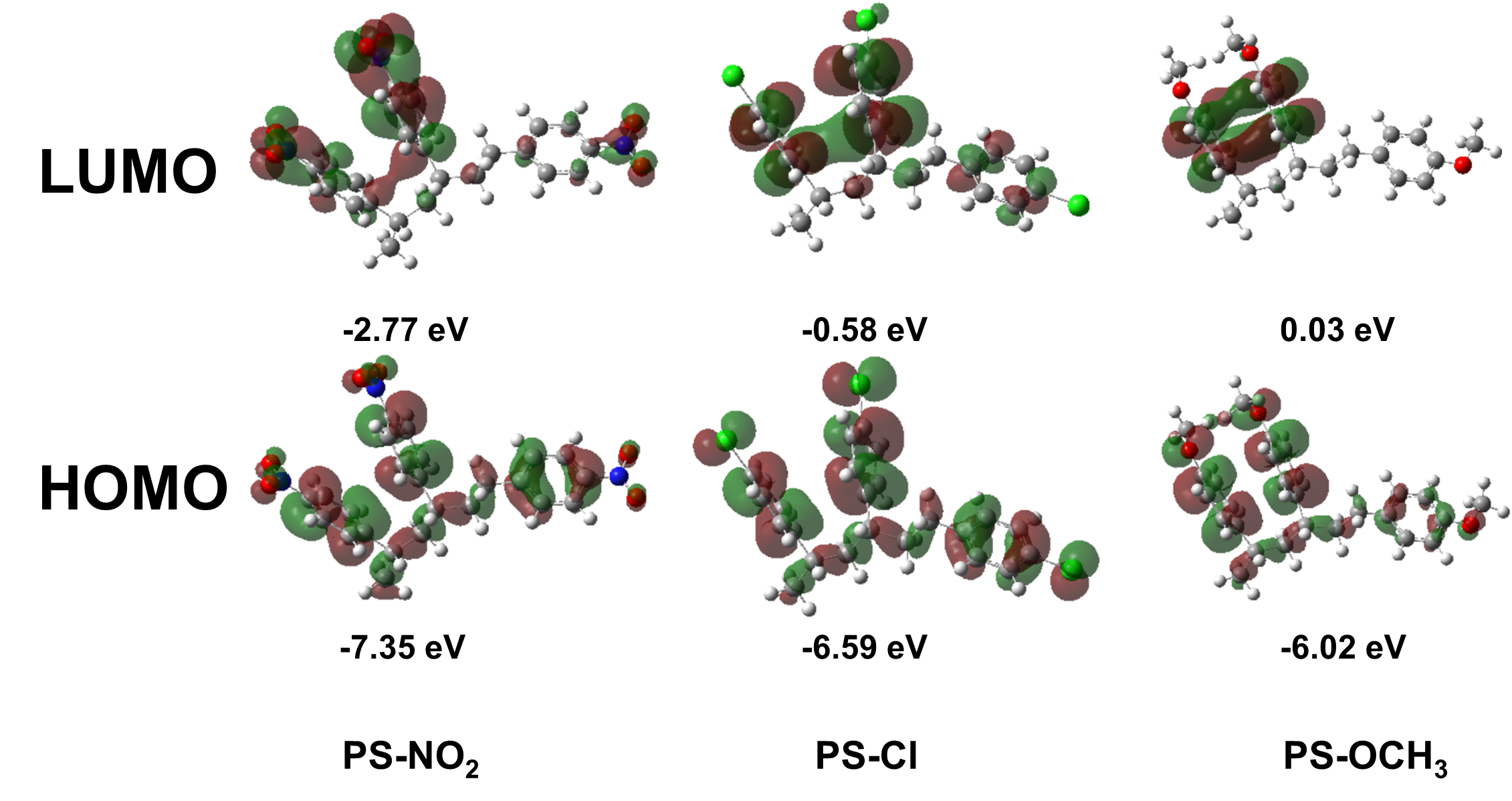


**Figure S9** The energy level of substituted polystyrene homopolymers.

**Figure S10.** The *D-E* loops of PS with different fileds at room temperature.

**Figure S11.** The *D-E* loops of PS-NO_2_ with different fileds at room temperature.

**Figure S12.** The *D-E* loops of PS-Cl with different fileds at room temperature.

**Figure S13.** The *D-E* loops of PS-OCH_3_ with different fileds at room temperature.

| **Entry** | **XSt (St)/MMA in molar ratio** |
| --- | --- |
| MS | 53/47 |
| MS-NO_2_ | 56/44 |
| MS-Cl | 52/48 |
| MS-OCH_3_ | 52/48 |

**Figure S14.** The ^1^H NMR characterization and the molar ratio of copolymers.


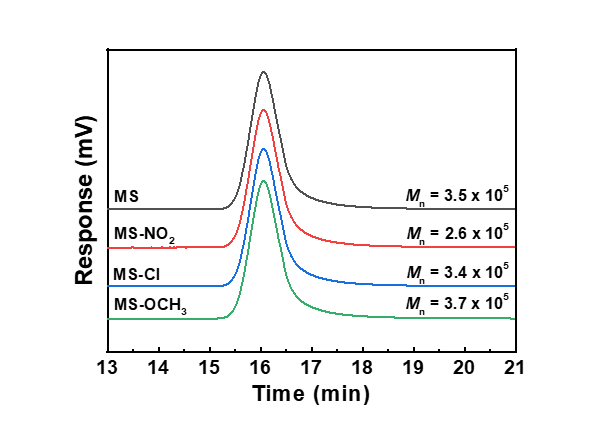


**Figure S15.** The GPC characterization of copolymers.


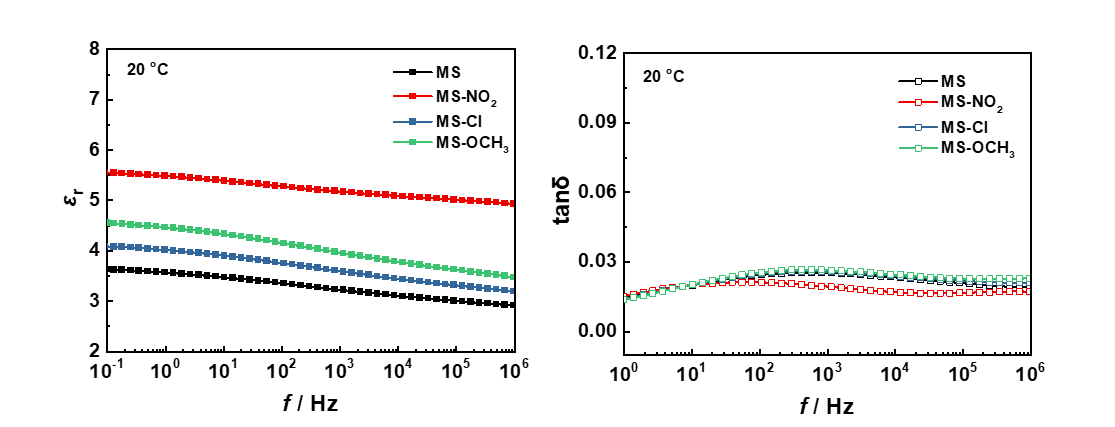


**Figure S16.** The dielectric constant and loss factor of the polymer as a function of frequency.

# Author Contributions

The manuscript was written through the contributions of all authors. All authors have given approval to the final version of the manuscript. Yipin Cheng and Honghong Gong are co-first authors.

Yipin Cheng: data curation (lead), formal analysis (lead), investigation (lead), software (lead), validation (lead), visualization (lead), writing - original draft (lead), writing - review & editing (lead); Honghong Gong: formal analysis (lead), investigation (equal), project administration (equal), writing - review & editing (equal); Meirong Zhang: investigation (equal), project administration (equal); Qinglong Ji: investigation (equal), project administration (equal); Guanxiang Zhang: investigation (equal), project administration (equal); Xiao Zhang: investigation (equal), project administration (equal); Zhicheng Zhang: formal analysis (lead), funding acquisition (lead), investigation (equal), project administration (equal), writing - review & editing (equal).
